# Job-search strategies of individuals at risk of poverty and social exclusion in Spain

**DOI:** 10.1371/journal.pone.0210605

**Published:** 2019-01-11

**Authors:** María José Gómez-Torres, Javier Rodríguez Santero, Javier Gil Flores

**Affiliations:** 1 Department of Didactics and Educational Organization, Faculty of Educational Sciences, University of Seville, Seville, Spain; 2 Department of Research Methods and Diagnostics in Education, Faculty of Educational Sciences, University of Seville, Seville, Spain; Universita degli Studi di Bari Aldo Moro, ITALY

## Abstract

In Spain, the issue of unemployment or precarious employment worsen with globalization, leading to an expansion of the so-called working poor in the labour market. According to previous literature, the economic poverty that is characteristic of this group may accompany poverty competency. In particular, the working poor resort to informal and poorly developed job-search strategies. This study addresses the job search methods used by people at risk of poverty and social exclusion. It provides evidence on the subject and serves as a basis for the adaptation of socio-labour intermediation programmes to this group. The hypothesis of this study is that people at high risk will predominantly use informal strategies that require a low level of job-search skills. A survey-based correlational study is conducted using a questionnaire completed by 279 people participating in socio-labour intermediation programmes developed by the Action against Hunger Foundation (AaHF) in Spain. Information on poverty indicators and on job-search strategies is collected. Data are analysed through cluster analysis, which distinguish two groups of people at risk of poverty and social exclusion (high risk and low risk), followed by a comparison of means (t-test) with a subsequent calculation of effect size using Cohen's d. Results show statistically significant differences with a medium effect size (between 0.45 and 0.50) for the typology of job-search strategies used, confirming the initial hypothesis. These results offer relevant information that should be considered when developing programmes aimed at improving social and labour issues for people at risk of poverty and social exclusion.

## Introduction

Spain’s employment data [1] reflect a reality marked by high unemployment and precarious, temporary and seasonal employment. This reality calls into question the veracity of the economic recovery announced by political and administrative bodies and encourages discussion of the traditional role of paid employment and whether it is an effective measure to avoid poverty and social exclusion [2,3]. These circumstances lead to the proliferation in the labour market of a social category known as the working poor, that is, workers at risk of poverty and social exclusion [4]. In Spain, the working poor total 14% of the population in 2016 [3] primarily consists of young individuals and individuals over 55 years of age [5].

The working poor are the result of the effects of economic globalization [6–11]. These effects include economies that have been stagnant for long periods, the presence of large cohorts of unemployed young individuals with low qualifications, the incorporation of the female labour force into the labour market, the lengthening of the working career along with the presence of older workers, an increase in the number of immigrants, low wages, inflated prices for basic goods, cuts in benefits and social services, unceasing demand for workers with high professional qualifications in the face of low demand for low-skilled workers, difficulties for small businesses in entering emerging markets and difficulties for the self-employed who wish to take advantage of new employment niches [6].

Workers who cannot satisfy basic vital needs with the salary they receive or avoid poverty during periods of temporary unemployment [12] are an increasing presence in our communities. This phenomenon is a cause for alarm for society because it indicates that work no longer represents a way out of poverty as it did in the past. In contrast, evidence suggests a relationship between a high level of intelligence and a favourable economic situation [13,14]. This argument can also be extrapolated to the national level given the association between intelligence and a country’s economic growth [15–17]. Consequently, it can be affirmed that there is a negative relationship between intelligence and poverty [18–21].

The globalization model that underpins current economic development is causing an increase in social inequality [22–27]. The seventh European Anti-Poverty Network Spain (EAPN-ES) report (2017) [3] analyses the situation in Spain using the European at-risk-of-poverty and/or exclusion (AROPE) indicator, which measures the risk of poverty and social exclusion of citizens of European Union countries. The report concludes that the wealthiest 10% of the Spanish population receives the same total income as 50% of the population. The AROPE indicator is constructed based on three criteria proposed by the Statistical Office of the European Union (Eurostat) in the framework of its Europe 2020 Strategy: the poverty risk rate, a severe lack of material goods and belonging to households that present very low labour insertion. A person is at risk of poverty and/or social exclusion when he or she meets at least one of the three criteria, which are described in greater detail below [28].

a) The poverty risk rate or income level below the poverty line (after accounting for the social transfers received) is calculated annually from the income obtained the previous year and is set at 60% of the median of the gains or rents per consumption unit. The EAPN-ES report indicates that in 2016, more than 2.9 million Spaniards live below this threshold (approximately 6.4%). The total income per consumption unit in these households is less than € 4,101 per year (€ 342 per month).

A person is in a situation of social exclusion when that person is trapped outside the social processes and structures of the community. Paradoxically, this group of socially excluded individuals is outside the system that cause the situation of vulnerability and social risk. The EAPN-ES report indicates that despite the macroeconomic classification in Spain, the 10,382,000 Spaniards affected by poverty constitute the highest percentage of the entire historical series (22.3%). This group is the most vulnerable to the effects of the crisis and requires a longer period to overcome poverty.

b) According to the European Survey on Income and Living Conditions (EU-SILC) for 2017, severe material deprivation characterizes households that cannot satisfy at least four of the following needs: paying housing-related bills, maintaining an adequate temperature in the house during the winter months, being able to cope with unforeseen expenses, eating meat or fish every two days, taking a vacation of one week a year, owning a car, owning a washing machine, owning a colour television and owning a telephone. The EAPN-ES report indicates that the groups with the greatest need during 2016 are women, single-parent households, young individuals (between 16 and 29 years old) and the foreign population.

c) A low intensity of employment in households occurs when members of the family unit of working age have been employed for less than 20% of the total annual time that could have been devoted to work. The EAPN-ES report notes that 14.9% of the population aged between 0 and 59 years lives in households with very little employment (5,265,263 individuals). The most affected groups are individuals between 45 and 65 years of age (20.1%) and individuals between 16 and 29 years age (17.6%), with no significant differences according to sex.

Similar to the 2016 EAPN-ES report, a report presented in January 2018 by Oxfam Intermón [29] places the poverty rate in Spain at 22.3%. Thus, Spain (along with Lithuania) represents the European country with the third-highest level of inequality after Romania and Bulgaria. Spain’s inequality is a consequence of the high profits earned by Spanish corporations (200.7%). In comparison, the cost per worker in Spain has only increased by 0.1%. In addition, the productivity per worked hour has increased by 6% (which favours the corporations), while the cost per hour of work of a worker has increased by less than 0.6%. For young individuals and women the situation is even worse. A total of 54% of young individuals are employed on part-time contracts, and women suffer salary inequality of up to 14% compared to men in the same job.

Because of the so-called “textual silence on poverty” (pg. 313 in [30]), few scholars have focused on the impoverished in studies that address the circumstances of minorities, the disadvantaged and/or marginal groups. The multiple factors that define the risk of poverty and social exclusion [31] (e.g., economic, labour, cultural, political, social, personal, environmental and housing) indicate the existence of forms of poverty other than the economic form [32]. For example, poverty of competencies highlights the importance of education in escaping social exclusion [33]. This type of poverty marginalizes individuals by affecting their opportunities for life-long learning, hindering their access to quality employment and limiting their participation in the agency of power and decision-making. Thus, it hinders people’s ability to take control of their lives and make decisions that directly concern them [34]. It also affects individuals’ attitudes towards employment, a factor that substantially influences their working life much more than other factors, such as age, sex, marital status, educational level and psychosocial and demographic characteristics [35,36]. These attitudes play a fundamental role in the employment of workers (employed or unemployed) and their advancement because they influence job-search behaviour [37,38] and the importance of insertion and reintegration into the workplace for adults [35,39–44].

There is a link between situations of social vulnerability and the job-search strategies used [45–47]. Consequently, non-profit entities develop training and social-labour intermediation programmes aimed at the most defenceless people in the labour market. These programmes seek to foster positive attitudes towards work and improve the skills of participants in the search for employment by teaching strategies that enhance the likelihood of insertion or re-employment [40,48], taking into account the fact that “people living in poverty tend to have inadequate job search skills” (pg. 360 in [49]). In addition, such programmes complement the knowledge and skills that participants already possess while encouraging them to continue looking for work. The counsellors of these programmes can transform the demoralization and apathy caused by unemployment (or precarious or unwanted partial work) into a proactive attitude towards employability if they understand their trainees’ attitudes towards employment and the job-search strategies they use to achieve labour insertion.

“Job search” is defined as “the behavior through which efforts and time are expended to acquire information about labor market alternatives and to generate employment opportunities” (pg. 129 in [50]). Two basic types of job-search strategies are identified: 1) formal methods represented by the use of, for example, recruitment agencies, employment offices, temporary employment agencies, the media (e.g., the press), Internet portals and job boards and 2) informal methods, which consist of networks of personal contacts (i.e., family and friends), networks of professional contacts (i.e., colleagues, former clients and suppliers) and self-candidacy (a curriculum vitae, self-employment and preparation for competitive examinations for public employment) [45,51–54].

Generally, although not exclusively, informal methods are the most commonly used and represent the main resource of individuals who belong to disadvantaged, marginal and minority social groups [43,55], such as women [45,52], immigrants [55,56] and blacks [57]. Although such methods represent an effective and quick approach to finding employment, they are more segregating than formal methods for two reasons [52]. First, the personal and family contacts a jobseeker approaches may be employed in jobs that discriminate for reasons of gender, race or nationality. Second, informal employment networks are subjective and favour the selection of individuals of the same gender, race and condition, thus perpetuating the labour segregation of these groups. In contrast, formal methods offer advantages because “the formal methods are in an open job market that anyone can learn about. Inside an organization, internally posting jobs notifies all workers of open positions. Similarly, newspapers, newsletters, and journals are fair mediums through which employers announce openings” (pg. 333 in [52]).

Both employed and unemployed workers combine different channels and methods of job searching [48,50,54,55,58,59], although the most educated people, who have higher levels of training and professional qualifications, use a greater number of strategies simultaneously and use predominantly formal methods over informal methods [58]. The Internet stands out as a job-search tool [41,50,53,54,59–64] because it succeeds in smoothing markets and eliminating physical and geographic barriers. In addition, it facilitates the elimination of hierarchies that control access to information to all subjects without intermediaries. However, to be a practical tool in the employment search, the Internet requires network access and the ability to use the Internet. Individuals who do not meet these requirements risk falling into a so-called digital divide that prevents them from accessing new segments of the labour market associated with Internet use. Thus, a new profile of the individual in search of employment has emerged: the Internet job seeker. This individual is a young person, typically younger than 35 years, who is a regular user of social networks, who has a high level of education and who is probably unemployed or looking for his or her first job [61].

In this study, the job-search strategies that characterize individuals at risk of poverty and social exclusion in Spain are examined. Specifically, the study aims to answer the following research question: what differences exist among job-search behaviours developed by individuals at different levels of risk of poverty and social exclusion? Based on previous empirical evidence [37,43,45,52,55,56,58], the starting hypothesis is that people at high risk of social exclusion make use, to a greater extent than people with low risk, of informal strategies that require a low level of job-search skills. Identifying the strategies to which lesser-experienced individuals resort is relevant information for the services and other entities that endeavour to assist this group. This information can help to focus the activities of such services and entities in the area of guidance and empowerment for social-labour insertion. Such information should be of particular help to those who desire to develop the skills and strategies of job seekers by providing training tailored to their needs.

## Method

In this study, it is adopted a non-experimental *ex post facto* research design based on survey methods. A correlational approach is used to provide evidence regarding the relationship between the level of the risk of poverty and social exclusion and job-search behaviour. The study has been reviewed and approved by the Governing Council of the University of Seville and complied with the ethical requirements for this type of study.

### Participants

A total of 279 people of legal age participate in the study. All of them complete an anonymous questionnaire in which they are asked to give their express written consent so that their answers could be used in the study (in application of article 6.1 of Organic Law 15/1999 of 13 December on the Protection of Personal Data corresponding to Spanish Legislation).

Participants are selected if they has taken part in socio-labour intermediation programmes developed by the Action against Hunger Foundation (AaHF) in various Spanish autonomous communities (Andalusia, Castilla-La Mancha, Catalonia, Extremadura, Galicia, Madrid, Murcia and Navarra). These programmes aim to facilitate access to employment and improve the employability of individuals at risk of social exclusion. In the sample, 60.6% of the participants are women, and 39.4% are men. A total of 15.2% are between the ages of 16 and 29 years, 50% are between 30 and 44 years, and those aged 45 years or older represent 34.8%. Most of the participants are Spanish citizens (89.0%) with a minority with dual (1.5%) or foreign (9.5%) nationality. Regarding the level of training that it is achieved by the studied sample, 24.8% has attended university studies, while 16.3% has only primary education and 2.4% has no education. Finally, 83.5% of the surveyed individuals indicate that they are unemployed.

### Variables and instrument

The first group of variables corresponds to various risk indicators of poverty and social exclusion. This group includes various variables related to economic capacity, services available and used at the home and consumption habits (Table 1). All these variables are measured in a dichotomous way, with the value of 0 when the implicit condition in the variable’s definition is not present and 1 when it is present. The data corresponding to these variables are collected through a questionnaire administered to the participants (the protocol used can be consulted at dx.doi.org/10.17504/protocols.io.uxdexi6).

**Table 1:** Variables and descriptive statistics.

Variable	Descriptive statistics
*Poverty risk indicators*	
Annual income less than € 8,000	60.2%
Inability to pay bills (e.g., gas, electricity, water, telephone)	53.9%
Inability to face unforeseen expenses	65.6%
No Internet connection at home	21.5%
Does not use heating in the home	37.3%
Does not consume meat and fish weekly	31.7%
Do not take a vacation of at least one week a year	73.5%
*Job-search strategies*	
Visit companies in search of work	M = 4.94; SD = 3.45
Call someone asking for work	M = 4.99; SD = 3.59
Spread the word that you are looking for work	M = 6.99; SD = 3.17
Search for information on the labour market	M = 7.33; SD = 2.97
Consult employment offices	M = 6.08; SD = 3.63
Place ads offering to work	M = 4.58; SD = 3.83
Study / train in subjects related to one’s own professional specialty	M = 6.44; SD = 3.56
Contact professionals in the same field	M = 6.01; SD = 3.57
Perform temporary work	M = 4.87; SD = 3.87

M = mean. SD = standard deviation.

In addition, information about job-search strategies is collected. For this purpose, the subscale “Job-search style” is used, which is included in the “Attitudes towards employment scale” [39,44]. The subscale consisted of nine items related to different ways of approaching the job search (Table 1). The value given to these variables ranged from 1 to 10 depending on the frequency with which the indicated activities are performed. In the application of this instrument [42] to a sample of 262 individuals registered in the employment services of the Autonomous Community of Andalusia, the reliability measured by Cronbach’s α is 0.854. In this study, it is obtained a reliability index of α = 0.823.

Table 1 contains the list of variables used, including basic descriptive statistics. For the indicators of risk of poverty and social exclusion, percentages of prevalence are shown. For the job-search strategies, means and standard deviations are included.

### Data analysis

The study starts with a cluster analysis of the participants based on the values obtained for the poverty risk indicators. Ward’s method of aggregation is used, and as a measure of proximity between the cases, the Euclidean distance applied to dichotomous variables is adopted. The results of this classification facilitate the identification of two groups of individuals. Differences between the two groups are found in terms of the values obtained for the indicators of risk of poverty and social exclusion. The differences between the groups with respect to these indicators are contrasted using the chi-square test. Finally, the differences between the job-search strategies used by individuals with a lower risk of poverty and social exclusion and those with a greater risk are analysed. The t-test is used for the contrast of means, with subsequent calculation of the effect size using Cohen’s d.

## Results

### Classification according to risk of poverty and social exclusion

**Fig 1:**
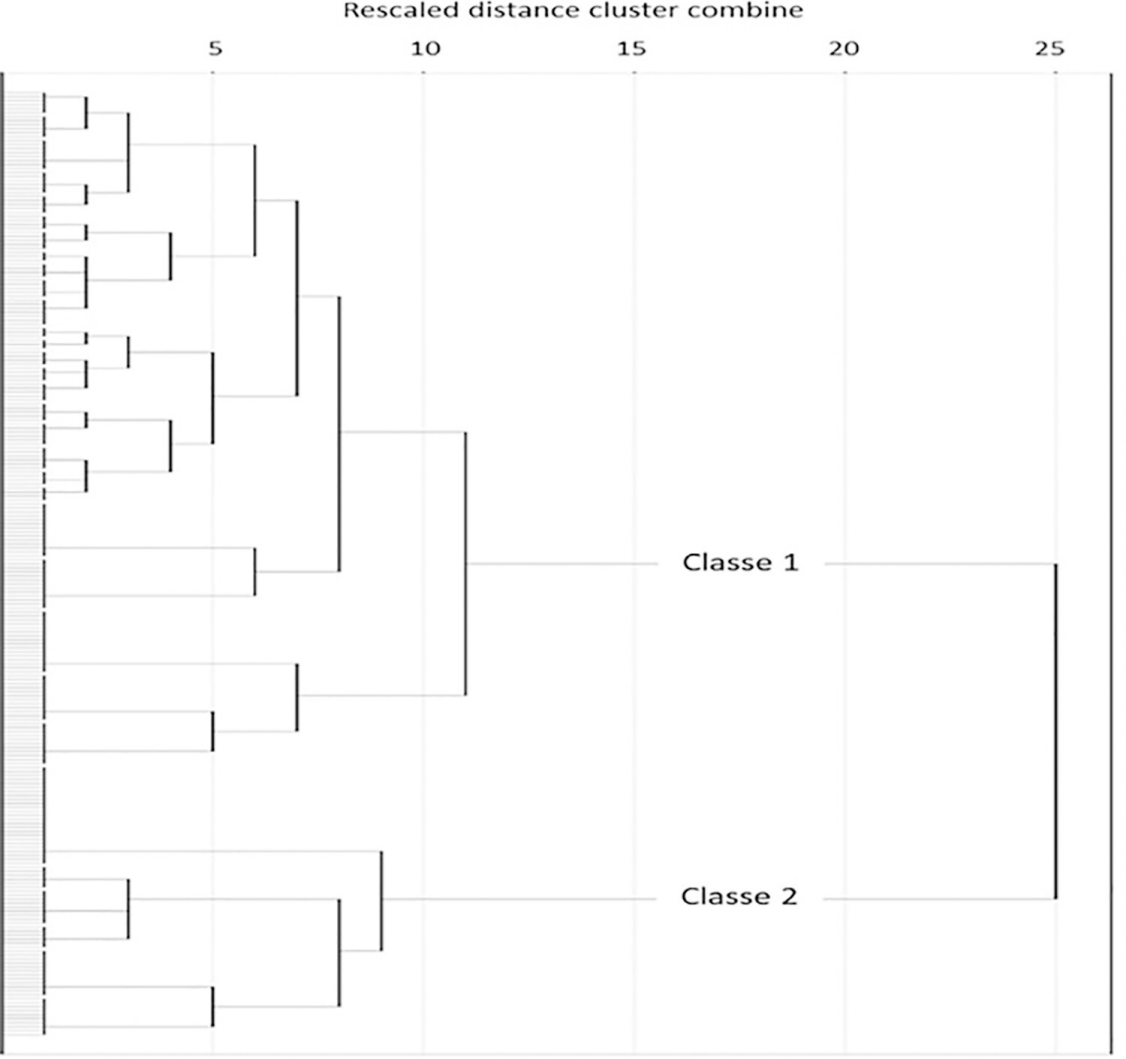
Dendrogram for classification according to risk of poverty and social exclusion.

Using cluster analysis, the Ward method and the Euclidean distance, it is obtained a hierarchical classification of the participants. A dendrogram (Fig 1) that corresponds to this classification shows the existence of two conglomerates that join a rescaled distance of 25 compared to the remaining conglomerates, for which the distances are equal to or less than 10. Partitioning the sample into two classes resulted in two groups constituted by 71.3% and 28.7% of the participants, who are differentiated by their situation in relation to the different risk indicators of poverty and social exclusion.

For each of the two resulting classes (Table 2), the percentage of individuals for whom each of the risk indicators of poverty and social exclusion is present is established. According to these results, a high percentage of those who constituted Class 1 receives incomes less than € 8,000 in the previous year (74.0%), can not pay bills for basic services in their homes (75.7%) or can not afford unexpected expenses (85.2%). The percentage of individuals in Class 2 who is in these circumstances is considerably lower, and none of them is unable to pay bills. Additionally, no member of Class 2 is unable to eat meat or fish weekly or is compelled to forgo heat, circumstances that affect approximately half of those included in Class 1 (45.0% and 51.1%, respectively). A total of 42.6% of the individuals included in Class 2 can not take a vacation of one week a year, whereas in Class 1, this percentage is more than double (85.8%). The differences are statistically significant. According to these results, Class 1 can be identified as individuals at a high risk of poverty and social exclusion, whereas Class 2 corresponds to individuals for whom this risk is considerably lower.

**Table 2:** Characterization of the classes based on the risk indicators of poverty and social exclusion.

Poverty risk indicators	Class 1 (%)	Class 2 (%)	Chi-square test
Annual income less than € 8,000	74.0	29.4	**
Inability to pay bills (e.g., gas, electricity, water)	75.7	0.0	**
Inability to face unexpected expenses	85.2	14.7	**
No Internet connection at home	27.8	8.8	*
Does not use heating in the home	52.1	0.0	**
Does not consume meat and fish weekly	45.0	0.0	**
Does not take a vacation of at least one week a year	85.8	42.6	**

* p < 0.01

** p < 0.001.

### Job-search strategies according to the risk of poverty and social exclusion

In this study it is compared (Table 3) the use of job-search strategies by individuals with high and low risks of poverty and social exclusion. The results indicate that in general, the strategies most used by participants are searching for information on the labour market (average of 7.55 for individuals with high risk and 7.03 for those with low risk) and spreading the word that one is looking for work (means: 7.27 and 6.33, respectively), while the least used are placing ads offering to work (means: 5.06 and 3.20, respectively) and performing casual jobs (means: 5.14 and 3.41, respectively). All of the considered strategies are most frequently used by those who are at high risk of poverty and exclusion. Based on the t-test results, these differences are statistically significant for four of the considered strategies. The largest differences appeared with respect to placing ads offering to work (t = 3.25, p < 0.01), performing casual work (t = 2.97, p < 0.01) and calling someone to ask for work (t = 2.01, p <0.01). The effect size for these differences is at medium levels, with Cohen’s d values between 0.45 and 0.50. Less important is the difference with respect to the strategy of spreading the word that work is being sought, for which a value of t = 1.99 (p < 0.05) with a low effect size (d = 0.30) is obtained. In short, the strategies that individuals at high risk of poverty and social exclusion use more frequently than those at low risk are informal strategies that are more immediate and require less development in job-search skills: performing temporary jobs, placing ads, asking someone for work and spreading the word. In contrast, formal strategies, such as going to employment offices, visiting companies, training in a specialty or consulting with professionals in the sector, are not common in individuals at high risk of poverty.

**Table 3:** Comparison of job-search strategies between individuals with high and low risk of poverty and social exclusion.

	High risk		Low risk		t-test	Cohen’s d
	Mean	SD	Mean	SD		
Visit companies in search of work	5.22	3.47	4.35	3.41	1.65	0.25
Call someone asking for work	5.38	3.61	3.82	3.36	2.91**	0.45
Spread the word that you are looking for work	7.27	3.04	6.33	3.25	1.99**	0.30
Search for information on the labour market	7.55	2.88	7.03	2.89	1.18	0.18
Consult employment offices	6.34	3.54	5.31	3.64	1.90	0.29
Place ads offering to work	5.06	3.80	3.20	3.63	3.25**	0.50
Study / train in subjects related to one’s own professional specialty	6.62	3.56	6.16	3.52	0.84	0.13
Contact professionals in the same field	6.38	3.52	5.35	3.61	1.90	0.29
Perform temporary work	5.14	3.90	3.41	3.68	2.97**	0.46

* p < 0.05

** p < 0.01.

## Discussion and implications

The findings of this study enable to draw two basic conclusions. On the one hand, the results reveal the existence of two groups of individuals at risk of poverty within the studied sample: one group with a high risk of poverty and another with a low poverty risk. On the other hand, the results also enable to answer the research question that constitutes the basic objective of the study: what differences exist among the job-search behaviours of individuals at different levels of poverty and social exclusion risk?

As indicated, in the studied sample, it is possible to identify two different profiles among the users of the programmes developed by the AaHF. Although all participants in these programmes are at risk of poverty and social exclusion, there is evidence that there is a group at high risk of poverty and another at low risk. This differentiation is important because, as showed earlier, both groups exhibit statistically significant differences with respect to job-search strategies. Thus, results indicate that the high risk of poverty group resorted to a greater extent (than the lower risk group) to informal methods that require few skills to search for employment, such as performing temporary jobs, posting ads, asking for work from family or friends and spreading the word regarding their unemployment. Though, the nature of the study does not allow to draw conclusions about the direction of this relationship that has been explored only through correlations.

However, results go in the direction of previous studies [50–57] showing that marginal, minority and disadvantaged groups tend to use informal job-search strategies and have limited access to quality jobs [52]. This relationship indicates that these individuals are more likely to work in low-skilled jobs with a high segregating character that are poorly paying and that offer little possibility of promotion. In contrast, formal job-search strategies provide advantages for minority groups because “for women and outsider groups, acquiring credentials and then using formal methods in the job search may assist in finding work in less-segregated workplaces” (pg. 333 in [52].

Considering the previous results, the following recommendation can be made. To escape poverty and social exclusion, job seekers must consider that the higher the levels of competitiveness they achieve in the formal employment market are, the more options they have to find quality jobs. In fact, formal methods are those most frequently used by employers more committed to the equitable treatment and diversity of their workers because “companies would probably not spend money or time on a formal job search for a bad job that virtually anyone could fill. Thus, companies may formally advertise only positions that are relatively advantaged as compared to positions requiring little skill or experience. Those companies and supervisors who use formal procedures for hiring may also be more committed to diversity” (pg. 334 in [52].

Results coming from the study contributed to give precious indications for counsellors and policy makers. Results could help them adapt their programme objectives and training activities to the socio-labour needs of individuals at risk of poverty and social exclusion who participate in such programmes. Formal job-search strategies should be promoted in socio-labour intermediation programmes and in guidance for employment. Such strategies guarantee access to jobs that are inclusive, high-quality and better remunerated and that offer better prospects.

This study can also be considered an accurate portrayal of the current Spanish labour market. It reflects the high percentage of workers in precarious situations, including those in part-time and/or seasonal employment against their preference, which is characteristic of the Spanish labour market. In addition, the predominant presence of women in the sample reflects the proactive attitude of this group, which has been particularly affected by the employment crisis and socio-labour vulnerability in Spain.

However, the study is very specific sample makes it difficult to generalize these results. Thus, it would be interesting to validate the results with larger samples in which random sampling procedures are used. To this end, the study could be extended to other European countries in which the AaHF is pursuing its orientation initiatives and socio-labour intermediation with individuals at risk of poverty and social segregation. New collaboration agreements could also be established with other entities and organizations that are similarly engaged.

Finally, from a theoretical point of view, this study contributes to knowledge on the strategies that individuals at risk of poverty and social exclusion adopt in the search for employment. Although this issue has been addressed in previous studies, the present work provides evidence obtained in the Spanish context, a setting not previously examined by other studies. In Spain, it has been shown that there are groups that are at different levels of risk and that this characteristic conditions the choice of the strategies used in the employment search.

## Supporting information

S1 Dataset Research data. (XLSX)
